# Age-Related Decline in Associative Learning in Healthy Chinese Adults

**DOI:** 10.1371/journal.pone.0080648

**Published:** 2013-11-12

**Authors:** Annie Lee, Jo Archer, Caroline Kai Yun Wong, Shen-Hsing Annabel Chen, Anqi Qiu

**Affiliations:** 1 Department of Biomedical Engineering, National University of Singapore, Singapore, Singapore; 2 Division of Psychology, Nanyang Technological University, Singapore, Singapore; 3 Clinical Imaging Research Centre, National University of Singapore, Singapore, Singapore; 4 Singapore Institute for Clinical Sciences, the Agency for Science, Technology and Research, Singapore, Singapore; University Of Cambridge, United Kingdom

## Abstract

Paired associates learning (PAL) has been widely used in aging-related research, suggesting an age-related decline in associative learning. However, there are several cognitive processes (attention, spatial and recognition memory, strategy, and associative learning) involved in PAL. It is unclear which component contributes to the decline in PAL performance associated with age effects. The present study determines whether age effects on associative learning are independent of other cognitive processes involved in PAL. Using a validated computerized cognitive program (CANTAB), we examined cognitive performance of associative learning, spatial and recognition memory, attention and strategy use in 184 Singaporean Chinese adults aged from 21 to 80 years old. Linear regression revealed significant age-related decline in associative learning, spatial and recognition memory, and the level of strategy use. This age-related decline in associative learning remains even after adjusting for attention, spatial and recognition memory, and strategy use. These results show that age effects on associative learning are independent of other cognitive processes involved in PAL.

## Introduction

Visual paired associates learning (PAL) administered with Cambridge Neuropsychological Test Automated Battery (CANTAB) assesses visual memory and new learning. The visual CANTAB PAL has been widely used in studying age-related memory decline and early detection of possible dementia [1]–[7]. It has also been employed as an assessment tool to evaluate the effectiveness of new therapies for dementia [8]. While the test continues to be widely used in aging-related research, it remains largely unknown whether age effects on associative learning are independent of other cognitive processes involved in the visual PAL, such as spatial and recognition memory, attention, and strategic process.

Spatial and item recognition memory was identified as a major cognitive process to be involved in the visual PAL [9]. Participants who are unable to recognize either an item or memorize its location would probably have difficulty in completing the visual PAL task. As age-related decline has also been observed in spatial and recognition memory [10]–[12], age-related decline in the visual PAL may not be caused predominantly by impairment in associative learning. As spatial recognition memory and pattern recognition memory have been found to be essential in associative learning, complementary tasks such as spatial recognition memory (SRM) and pattern recognition memory (PRM) can be included in the test battery to help isolate the main cause of any deficit in PAL performance [9].

The visual PAL may also require attention and sustained attention, which has been shown to decline with age [13]. Moscovitch (1994) [14] proposed a dual system hypothesis suggesting that the PAL is primarily sensitive to damage in both the medial temporal lobe area and the frontal lobe of the brain. Age-related decline has been shown in attention processes, mediated by the frontal lobe [15], that could be a potential factor in the age-related decline in associative learning performance [16]. Several studies have examined whether manipulating attention interferes with associative learning and have yielded conflicting results. Some studies showed that young adults performing paired association between unrelated non-word pairs (nonsense anagrams of real words) under divided attention had poorer performance than those performing under full attention [17], [18], whereas others reported no impairment under divided attention [19], [20]. Such differences may arise from the difficulty level of the verbal associative task as well as the type of secondary/distractor task employed across existing studies. Although all of these studies were conducted using non-word pairs where verbalization of stimuli is minimized, none used visual PAL. Hence, the role of attention in the PAL remains unclear. As attention could also potentially influence the interpretation of age-related decline in the visual PAL, it is important to consider this as a factor when interpreting impairment in associative learning.

Strategy may also affect the PAL performance, where strategy refers to a conscious effort to use a systematic strategic process to complete the task [21]. It has been shown that aging is associated with strategic rigidity assessed using the Wisconsin card sorting task (WCST) and older adults may be less efficient in engaging and applying appropriate strategies to enable them to successfully perform the WCST [22] **.** In studies of the strategic processes and associative learning, researchers investigated whether strategy use influenced the performance of associative learning tasks. For example, Dunlosky & Hertzog (1998) [23] collected strategy reports from participants during the verbal PAL testing and showed that three common types of strategies, including interactive imagery where image is created for each pair of stimuli, sentence generation where a specific sentence is created to integrate each pair of stimuli, and rote learning where each pair of stimuli is learned through repetition, could lead to differences in the performance of memory task recall. Participants who reported using interactive imagery and sentence generation during the associative task had better recall performance as compared to participants who employed rote learning. Nevertheless, further analysis in this study indicated that the relationship between age and memory recall was not influenced by types of strategies used, suggesting that the use of strategies may have minimal effect on the relationship between age and the PAL performance. Although the aforementioned results may indicate that strategy processes have a limited role in associative learning, Roger et al. [21] demonstrated that the usage of strategy could influence the relationship between age and associative learning if uncontrolled for. This may lead to a profound mediator effect on the interpretation of aging effect on associative learning [24], [25]. Few studies have attempted to examine the potential effect of strategic usage in associative learning [21], [23], [26] and all of these studies were performed using the verbal PAL where word-pairs could be verbalized. However, a recent validation study on the visual PAL has shown that the measures (the first trial memory score, total errors and total trials) appear to be strongly correlated with the verbal index of the Wechsler memory scale-revised (WMS-R) as compared to the non-verbal index of the WMS-R. Participants in the study also reported verbalizing the stimulus through attributing names to the displayed patterns in order to perform the Cambridge Neuropsychological Test Automated Battery (CANTAB) PAL effectively [27]. Given the paucity of data on strategy use in the visual PAL and the possibility that the visual PAL can be verbalized, it is possible that strategy choice may impact on the PAL performance, thus strategy is worthy of the investigation in aging studies on PAL.

Findings from the above studies suggest that spatial recognition memory, attention, and strategic processes are potentially confounding cognitive processes that may mislead the interpretation of a decline in associative learning in aging. To the best of our knowledge, no studies have simultaneously examined the influence of all of these cognitive processes when investigating the relationship between age and the PAL performance. In this study, we aimed to examine whether the age-related decline in associative learning is independent of the other cognitive processes, particularly those cognitive components proposed in the dual system model in a Singaporean Chinese population aged 21 to 80 years-old. This study employed the CANTAB visual PAL as the dependent measure. Three other CANTAB tasks were selected to represent the different components of the PAL: spatial working memory (SWM) providing both a spatial recognition score and a measure of strategy use; delayed match to Sample (DMS) representing pattern recognition, and intra-extra dimensional shift (IED) providing a measure of attention. We anticipated that performance on all of the cognitive tasks in this study would decrease with age. In addition, if associative learning is not the most sensitive component of the PAL to age-related memory decline, the relationship between age and PAL should be strongly influenced by the cognitive processes postulated in the dual system model.

## Methods

### Subjects

This study was approved by the National University of Singapore Institutional Review Board and all participants provided written informed consent prior to participation.

One-hundred and eighty-four healthy Singaporean Chinese volunteers aged 21 to 80 years old were recruited (males: 80; females: 104) for this study. The participants were recruited via advertisements and screened for eligibility through a phone interview prior to an onsite visit. Volunteers with the following conditions were excluded: (1) major illnesses/surgery (heart, brain, kidney, lung surgery); (2) neurological or psychiatric disorders; (3) learning disability or attention deficit; (4) head injury with loss of consciousness; (5) non-removable metal objects on/in the body such as cardiac pacemaker; (8) diabetes or obesity; (9) a Mini-Mental State Examination (MMSE) score of less than 24 [28]. Subjects’ characteristics are reported in Table 1.

**Table 1:** Demographics and Cognitive Measures.

Age Range	20s (n = 37) mean (SD)	30s (n = 27) mean (SD)	40s (n = 32) mean (SD)	50s (n = 46) mean (SD)	> 60 (n = 42) mean (SD)
Female, %	51.4	55.6	59.4	54.3	61.9
Age	25.8 (2.3)	34.0 (2.6)	44.4 (2.7)	54.4 (3.0)	67.1 (4.8)
Education	4.6 (0.6)	4.6 (0.6)	3.6 (1.1)	3.2 (1.0)	3.1 (1.3)
MMSE score	29.1 (1.0)	28.5 (1.4)	28.0 (1.4)	28.0 (1.5)	28.0 (1.5)
PAL -- first trial memory scores	22.0 (2.7)	21.0 (1.6)	19.0 (2.8)	18.3 (2.8)	17.3 (2.8)
IED – Pre-EDS errors	4.6 (1.4)	4.9 (2.2)	5.6 (2.1)	5.4 (2.0)	5.82 (2.6)
SWM – between search errors	12.6 (12.4)	12.5 (8.9)	17.1(14.2)	26 (17.4)	41.5 (20.5)
SWM -- strategy	30.8 (4.8)	29.3 (6.2)	31.8 (4.5)	34.7 (3.8)	37.1 (5.1)
DMS -- total correct for delayed recognition	17.9 (1.6)	17.5 (1.4)	16.7 (2.7)	17.2 (2.2)	15.9 (3.0)

*Abbreviations:* MMSE -- Mini-Mental State Examination; PAL -- Paired associates learning. IED -- Intra-Extra Dimensional Shift; DMS -- Delayed Matching to Sample; SWM -- Spatial Working Memory.

*Note:* Education scales: 0  =  no education, 1  =  primary school level, 2  =  secondary school level, 3  =  Singapore - Cambridge General Certificate of Education Ordinary Level (‘O’ level) / Singapore-Cambridge General Certificate of Education Normal (Academic) Level (‘N’ level), 4  =  Pre-University/Diploma/ITE/Certificate, 5  =  Degree and above.

Cognitive Tasks

The CANTAB consists of language-independent cognitive tests [29] administered on a computer fitted with a touch-sensitive screen and 2-button response pad. Participants were first screened on two motor and learning tasks to verify the ability to follow simple instructions. Subsequently, participants performed seven CANTAB tasks in this order: Intra-Extra Dimensional Set Shift (IED), Match to Sample Visual Search (MTS), Paired Associates Learning (PAL), Stockings of Cambridge (SOC), Stop Signal Task (SST), Spatial Working Memory (SWM) and Delayed Matching to Sample (DMS). Due to the long duration required to complete all of the selected tasks, participants were administered the IED, MTS, and PAL tasks and encouraged to complete the SOC, SST, SWM and DMS. As the focus of our current study was to examine the age-effects on the components within the PAL task, we only included tests (IED, SWM and DMS) with cognitive processes that more closely reflected these components. The DMS was chosen over MTS as it was thought that the DMS reflected the delay response aspect on top of a learning aspect involved in PAL. Furthermore, the PAL task is not testing for inhibition of prepotent response (measured by SST) and spatial planning (measured by SOC), hence neither task was included in the analysis. The detailed description of these tasks is provided below (demo of these tests can be found on the Cambridge Cognition's website: http://www.cantab.com). ****

A fixed order of tasks was used to ensure that similar tasks were placed apart in order to minimize the risk of subjects carrying aspects of the former task into the next task. In addition, the fixed order ensured the more demanding tasks were not presented consecutively so as to minimize fatigue.

**Intra/extra-dimensional shift (IED).** The IED task is an adaptation of the Wisconsin card sorting Test. It consists of two critical parts: maintenance of attention and shifting of attention (Laws et al., 2011). The IED involves a total of nine stages. In each trial participants are shown two abstract images, each comprised of a shape and an overlapping line. They are instructed to choose the correct image in accordance with an underlying rule. For each stage, six continuous correct responses are needed before the task moves to the next stage. If the six continuous correct responses are not obtained within fifteen trials at each stage, the task is automatically terminated. The number of errors committed on stage 1 indicates proficiency in detecting and learning the implicit rule of the task based on feedback as to whether the choice is correct. Stage 6 involves an intra-dimensional shift where shape remains the target cue, but the ‘correct’ shape changes. Stage 8 is the extra-dimensional shift (EDS stage) where participants must learn to shift attention from the previously correct dimension (the shape of the stimulus) to the newly correct dimension (the line). The number of errors made at the EDS stage indicates proficiency in extra-dimensional set-shifting. The total number of errors from stages 1 to 7, referred to as Pre-EDS errors, indicates proficiency in maintaining selective attention.

**Paired associates learning (PAL**). The PAL is a visuospatial associative learning task. Participants are presented with six white squares arranged in a circle. Patterns are sequentially presented in each of the six squares for three seconds in a random order. Subsequently, the same patterns are represented in the centre of the screen in a different order. The participants are instructed to select the square indicating the original location of the pattern. No time limit is enforced. The number of patterns presented increases if participants correctly locate the original location of every pattern on the first attempt. In the first stage only one pattern is presented, increasing to two patterns in stage 2, three patterns in stage 3, six patterns in stage 4 and finally eight patterns in stage 5. If participants fail to recall the locations correctly, the patterns in that stage are represented for up to 10 attempts per stage. Failure to recall the correct order after 10 attempts results in the termination of the task. The first trial memory score is calculated as the number of objects correctly associated to their locations in the first attempt for each trial in each stage. The first three stages consist of two trials with novel stimuli. Thus, in stage 1 the maximum points that a participant can be awarded are two, followed by four points for forming two correct object-location pairs in stage 2, then six points for forming three correct object-location pairs in stage 3. For stages 4 and 5, each consists of only one (novel) trial, and the maximum number of points awarded is six and eight respectively. The full score of the first trial memory for the task is 26 points. A higher score indicates better associative learning.

**Spatial Working Memory (SWM).** The SWM is a self-ordered searching task that requires participants to maintain and update spatial information in working memory. It has often been used to assess the heuristic strategy of participants. In each trial participants are asked to find blue tokens hidden in 3, 4 or 6 boxes. Within each trial, a token is hidden in one of boxes. They are told that the tokens are hidden one at a time and that over the course of one trial tokens are never hidden in the same box twice. Thus, participants need to make multiple searches within one trial: as soon as a token is found, a new search begins. A between-search error is scored when participants return to a box where a token has already been found and is used as the measure for spatial working memory. To reduce the load on working memory, participants can employ a pre-determined sequence strategy of searching. In other words, when participants found a token, they can restart the search using the sequence they previously used. The strategy score estimates the extent that this strategy is employed and gives an indication of the participant’s ability to use heuristic strategy. The range of the SWM strategy score is 1 to 37; higher scores indicate less evidence of strategy use.

**Delayed matching to sample (DMS).** The DMS assesses both simultaneous and delayed visual memory. In each trial, a complex visual pattern (the sample) is briefly shown (4.5 s). The sample is then covered and participants see four patterns below the sample after a delay of 0s, 4s or 12s. Participants are instructed to select the pattern identical to the sample. In this study, a DMS total correct delayed score is reported as the number of trials for which the participant selects the correct stimulus in trials of the delays of 4s and 12s, providing a measure of delayed recognition. The full score of this measure is 15. A higher score indicates better delayed recognition.

### Statistical analysis

First, we employed Pearson’s correlation analysis to examine the relationships between age and all of the cognitive measures: associative learning (first trial memory scores in the PAL); sustained attention (Pre-EDS errors in the IED); spatial working memory (between search errors in the SWM); strategy use (strategy score in the SWM), and delayed recognition memory (total correct for delayed recognition trials in the DMS).

Second, we examined the age-related decline in associative learning (the first trial memory scores in the PAL), sustained attention (Pre-EDS errors in the IED), spatial working memory (between search errors in the SWM), strategy use (strategy score in the SWM), and delayed recognition memory (total correct for delayed recognition trials in the DMS) using linear regression with age as the primary independent variable and cognitive measures as the dependent variable. Separate regression models were used for individual cognitive measures. Education level was entered as a covariate in all of the analyses as a categorical variable (1  =  primary school level, 2  =  secondary school level, 3  =  Singapore - Cambridge General Certificate of Education Ordinary Level (‘O’ level) / Singapore-Cambridge General Certificate of Education Normal (Academic) Level (‘N’ level), 4  =  PreUniversity/Diploma/ITE/Certificate, 5  =  Degree and above).

Lastly, we examined the relationship between age and associative learning using a multiple linear regression model where sustained attention (Pre-EDS errors in the IED), spatial working memory (between search errors in the SWM), strategy use (strategy score in the SWM), and delayed recognition memory (total correct for delayed recognition in the DMS) were entered as covariates. Finally, Fisher’s *z*-test was carried out to investigate whether the relationship between age and the associative learning was altered after adjusting the aforementioned cognitive measures when compared to that without adjusting them.

Although all the dependent variables in the above regression models were Gaussian distributed, the variance of the regression error terms in some of the above analyses was not constant for all values of age. Hence, robust standard errors were used to compute *p*-values for all aforementioned regression analyses to deal with heteroscedasticity. Bonferroni correction was applied to correct for multiple comparisons in this study.

All the data analysis was performed using SPSS 18 in Windows 7.

## Results

All 184 participants completed the IED and PAL tasks. Among them, only 103 participants completed the cognitive battery up to the SWM, and 85 completed the entire battery up to the DMS. Four participants were excluded from the PAL analyses because they aborted the task. A further eight participants were excluded from the IED analyses for the same reason. Logistic regression analyses indicated that the 85 participants who completed the entire cognitive battery were relatively younger than the entire cohort in this study (n = 184, OR = 0.97, p =  0.01).

Table 1 lists the mean and standard deviation of the demographics and the cognitive measures that were stratified in terms of decade. We further examined the interrelationships among these measures. Table 2 shows significant correlation of age with all of the cognitive measures. Additionally, with the exception of the IED measure, there were significant inter-correlations among the PAL, SWM and DMS measures.

**Table 2:** Correlation matrix among cognitive measures and age.

	Age	PAL – first trial memory scores	IED – Pre-EDS errors	SWM – between search errors	SWM – strategy	DMS – total correct for delayed recognition
Age	_	–0.544**	0.192*	–0.571**	0.478 **	–0.351*
PAL – first trial memory scores		_	–0.132	–0.485**	–0.309**	0.341**
IED – Pre-EDS errors			_	0.114	0.074	0.026
SWM – between search errors				_	0.682**	–0.401**
SWM – strategy					_	–0.388**
DMS – total correct for delayed recognition						_

* *p,*.01; **p,.001.

Abbreviations. PAL -- Paired associates learning; IED -- Intra-Extra Dimensional Shift; DMS -- Delayed Matching to Sample; SWM -- Spatial Working Memory.

**Figure 1:**
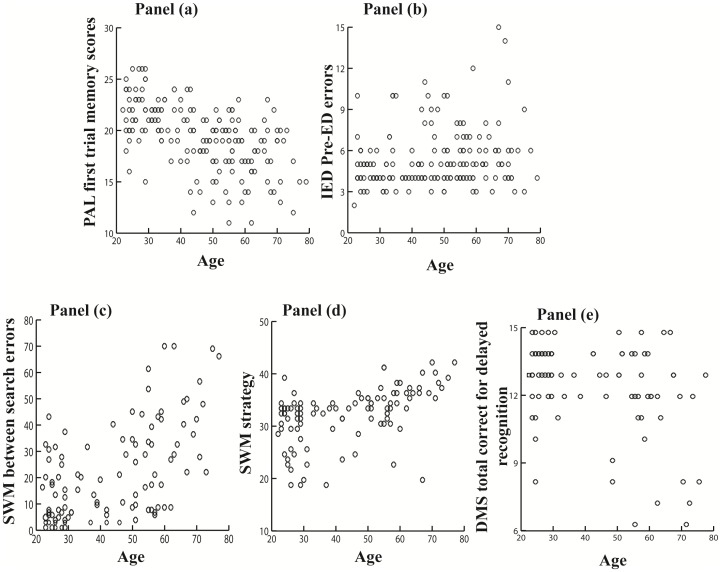
Scatter plots displaying the relation between CANTAB measures and age. Panel (a) shows the relation between PAL first trial memory scores and age (N = 180). Panel (b) shows the relation between IED Pre-ED errors and age (N = 176). Panel (c) shows the relation between SWM between search error and age (N = 103). Panel (d) shows the relation between SWM strategy and age (N = 103). Panel (e) shows the relation between DMS total correct for delayed recognition and age (N =  85).

Figure 1 shows the scatter plots of the cognitive measures in relation with age. Subsequent regression analyses revealed significant effects of age on the between search errors and strategy of the SWM task after adjusting for the education level (Table 3), indicating that as age increased individuals relied less on strategy use and made more between search errors in the spatial working memory task. A significant age effect was also found on the sustained attention measured by the Pre-EDS errors in the IED after adjusting for the education level (Table 3), showing age-related decline in sustained attention. However, this relationship did not survive correction for multiple comparisons. The significant association between age and delayed recognition memory score (measured by total correct score of delayed trials) was not statistically significant after adjusting for the education level.

**Table 3:** Relationships of cognitive measures with age.

	Sample Size	Mean (SD)	Standardised ß	*p-*value
PAL -- first trial memory scores	180	19.4 (3.1)	–0.49	<0.001
PAL -- first trial memory scores	85*	20.1 (3.4)	–0.63	<0.001
IED – Pre-EDS errors	176	5.3 (2.1)	0.19	0.029
SWM – between search errors	103	21.9 (18.6)	0.49	<0.001
SWM -- strategy	103	32.8 (5.4)	0.38	0.001
DMS -- total correct for delayed recognition	85	12.5 (2.1)	–0.24	n.s.

Abbreviations. PAL -- Paired associates learning; IED -- Intra-Extra Dimensional Shift; DMS -- Delayed Matching to Sample; SWM -- Spatial Working Memory; n.s. – not significant.

Note. All results are adjusted for education level; * includes only the participants who completed all CANTAB measures.

There was a significant negative association between age and first trial memory scores in PAL after adjusting for the level of education (p<0.001, Table 3), indicating that as age increases PAL performance decreases. This relationship remained unchanged in the 85 participants who completed the entire cognitive battery (p<0.001, Table 3). When further adjusting for sustained attention (Pre-EDS errors), strategy (SWM strategy), spatial working memory (SWM between search errors), and delayed recognition (total correct in the DMS delayed trials), age was still significantly associated with PAL performance (assessed by the first trial memory score) (p = 0.002, Table 4). Fisher’s z test indicated there was no significant difference in the relationship between age and PAL before and after adjusting for the other tasks (z  =  0.47; 1-tailed p  =  0.236).

**Table 4:** Age effect on the first trial memory in the PAL after adjusting for sustained attention (Pre-EDS errors), strategy (SWM strategy), spatial working memory (SWM between search errors), and delayed recognition (total correct in the DMS delayed trials).

	Standardised ß	*p*-value
Age	–0.42	0.002
IED – Pre-EDS errors	0.01	n.s.
SWM – between search errors	–0.21	n.s.
SWM -- strategy	0.13	n.s.
DMS -- total correct for delayed recognition	0.19	n.s.

Standardized ß-values and their corresponding p-values are reported below.

Abbreviations. PAL -- Paired associates learning; IED -- Intra-Extra Dimensional Shift; DMS -- Delayed Matching to Sample; SWM -- Spatial Working Memory; n.s. – no significance.

## Discussion

This study employed the CANTAB to assessed age-related changes in spatial and recognition memory, attention, strategy use, and associative learning. We also examined whether age-related decline in PAL is primarily due to impairment in associative learning, and not due to impairment in the other cognitive processes required in PAL.

There is a lack of norms for CANTAB tests in Asian populations, although these tests have been widely used in studies on aging and neurodegenerative diseases in UK samples [1, 2, 3, 9, 31 and 32]. In this paper, we presented the mean and standard deviation values of the CANTAB tests in each decade across lifespan, hence providing a useful reference for aging studies in the Asian Chinese population.

There was an age-related decline in the majority of the CANTAB measures used in this study (PAL first trial memory scores, IED Pre-ED errors, SWM between search errors and SWM strategy). Aging effects on PAL and SWM measures are consistent with the findings in normative CANTAB studies based on samples from Western countries [31], [32]. The decline of performance in PAL and SWM as a function of age suggested that memory for object-location could warrant concern especially when this form of memory is particularly crucial for daily activities in today’s multifaceted environment [30]. Although not a perfect comparison, among the cognitive measures presented here the standardized betas indicate that the PAL first trial memory score and SWM between search errors show a stronger age-related relationships. The stronger relationships between PAL and SWM measures and aging are consistent with the findings from other studies [31], [32]. Both tasks have been shown to be able to detect cognitive decline between the youngest group of the study, that is age bands of individuals below 55 years old, and those who are in the 55-59 years old subgroup, with high statistical power as compared to test of Stocking of Cambridge from CANTAB battery and Spatial Span Task, thus suggesting the usability of these two measures in detecting cognitive decline in older adults. In contrast to Robbins et al. (1998)’s [32] normative aging study in a Western sample, our results showed no association between age and delayed recognition memory assessed using DMS after adjustment for the education level. This difference could be explained by differing sample demographics, including age range and screening criteria.

Nonetheless, the selective age-related cognitive decline in spatial working memory (SWM between search errors) and strategy usage (SWM strategy) but not in sustained attention (IED Pre-ED) and delayed recognition memory (DMS total correct for delayed recognition) has two important implications. First, there were distinguished age effects on different forms of memory. For instance, pattern recognition memory is relatively spared from age-related decline compared to associative learning and spatial memory. This is consistent with a recent study exploring patterns and relationships of different forms of memory decline [33]. Silver, Goodman, and Bilker (2012) [33] investigated aging effects on logical memory, association memory, objection recognition memory, spatial memory, and verbal memory and found that among these memory processes, there was no significant difference between young and older adults in terms of object recognition memory. Second, selective age-related cognitive decline may provide indirect evidence of the brain regions involved in the different memory processes. Different forms of memory have been found to recruit distinct cortical regions, for instance, the hippocampus has been linked with associative learning and spatial memory tasks [34]. On the other hand, the perirhinal cortex has been implicated in object recognition, as lesions in the perirhinal cortex greatly impaired object recognition memory as compared to a lesion of hippocampus [35], [36]. In addition, the hippocampus suffers from considerable age-related changes whereas the entorhinal and perirhinal cortex are relatively spared from age-related degeneration [22], [37]. Taken together, these neurological findings may explain the behavioural age-related memory decline, though evidence for this is beyond the scope of this behavioural study.

In examining the influence of the cognitive processes in visual PAL, particularly the processes proposed by the dual system model, age effects on associative learning are found to be independent of other cognitive processes involved in PAL. This finding from our study renders some implications on the interpretation of the dual system model for PAL. The dual system model of PAL posits that the frontal cortex is an important component in the functional network involved in associative learning [14]. In this model, the medial temporal lobe/hippocampus is viewed as an unintelligent component that passively accepts input for storage purposes and provides information when cued to do so, while the prefrontal cortex is perceived as the intelligent component that organizes information according to their contexts and validates information before it is transferred to the medial temporal lobe/hippocampus. Additionally, a crucial part of this prefrontal system under the dual model lies in the initiation of strategies which in turn ensures that information presented during the task is encoded into the existing memories and retrieved effectively when needed. Associative learning would thus be thought to be dependable on the cognitive process in the frontal lobe to enable information to be organized before transference to the medial temporal lobe for storage. Hence, age-related decline in frontal mediated memory tasks such as SWM strategy and IED Pre-ED errors should have considerable effects on the CANTAB associative learning which requires complex association and remembering the arrangement of visual patterns presented. However, the findings from this study suggest that the age-related decline in the performance of associative learning is greater than that of frontal mediated tasks such as attention, spatial working memory and strategy use. Current results appear to reflect consistency with existing findings in which the medial temporal lobe/hippocampus component involvement in associative learning suggests the component itself is able to carry out complex association and arrangement of information. This consistency could be seen in recent study employing a similar PAL paradigm (i.e non-words pair). The study shows that performances for the visual PAL in subjects with mild cognitive impairment [32] was significantly lower when compared with healthy adults. Furthermore, fMRI studies also revealed that healthy adults had bilateral hippocampus activations as compared to adults with MCI despite similar frontal activation [38], showing the importance of the role of hippocampus in visual PAL.

There are however potential limitations to this study. First is the degree to which the chosen tasks represent the cognitive processes implicated in PAL; the neuropsychological tasks were chosen based on the currently available literature in both behavioral and neuroimaging studies. We selected tasks that could represent the different aspects of PAL as closely as possible. Furthermore, where possible the more difficult task was chosen to represent each component in order to be more conservative with the adjustment of that factor in the final adjusted model, hence the DMS task was chosen over the pattern recognition memory task in CANTAB. However, it is acknowledged that the tasks may not perfectly represent the aspect of the PAL task which they have been chosen to adjust for, or they may lack sensitivity. Secondly, the sample sizes across the cognitive tasks presented here are different. The sample sizes for the SWM and DMS were smaller as compared to the PAL and IED tasks. The absence of an association between DMS performance and age may be due to the smaller sample size and had the DMS sample size been similar to the full sample then there may have been a significant effect. Furthermore, even though the counterbalancing of the tasks is an important aspect of the experiment design, the cognitive tasks were examined in a fixed order in our study. In this study the cross contamination between tasks that are relevant to PAL was a major concern. Through customizing the tasks order, we ensured that similar tasks were placed apart from each other, i.e., MTS and DMS, and minimised the chances that subjects may carry aspects of the former task (instructions, irrelevant strategy,confusion over stimuli) into the next task. Nevertheless, we notice that this may increase the risk of a latent fatigue effect on the performance for later tasks, which could be avoided to have two or three sequences of tasks in the study design. Lastly, as we examined the role of attention as a general cognitive process, an absence of association between IED performance and age may be due to the task insensitivity to capture the specific attentional process needed in PAL. Future studies could attempt to differentiate the types of attentional processes in PAL.

In summary, this study showed that age-related findings explored in the UK population can be replicated using an Asian Chinese population. Additionally, the norms of the CANTAB tests in each decade across lifespan were presented. Lastly the study shows that PAL is also a sensitive cognitive test for aging in the Asian Chinese population and the effects of age on associative learning are independent of the other cognitive components involved in PAL.
